# Investigating the Effects of Epac2 Activation in an In Vitro Cortical Mechanical Injury Model for Central Nervous System Repair

**DOI:** 10.1111/ejn.70286

**Published:** 2025-10-25

**Authors:** Hongming Ma, Guy S. Bewick, Wenlong Huang

**Affiliations:** ^1^ Institute of Medical Sciences, School of Medicine, Medical Sciences & Nutrition University of Aberdeen Aberdeen Scotland UK

**Keywords:** central nervous system injury, Epac2, in vitro modeling, neuroprotection, S‐220

## Abstract

Globally, around 21 million people are currently living with a spinal cord injury (SCI), which causes loss of neural function and has no cure, creating substantial social and economic challenges. Several factors impede central nervous system (CNS) repair, including the limited intrinsic regenerative capacity of adult mammalian central nervous system neurons, the formation of cavities and glial scars, and the presence of inhibitory molecules at the injury site. Studies in an ex vivo SCI model suggest that exchange protein directly activated by cAMP 2 (Epac2) elevation by the agonist S‐220 can transform a post‐lesion inhibitory environment to one, which promotes axonal outgrowth. However, this ex vivo preparation did not allow the detailed and accurate assessment of responses of individual cell populations following injury. Moreover, it was unclear if S‐220 conferred neuroprotection in the ex vivo model. To address these issues, here we use a relatively novel but simple in vitro model of CNS injury to further examine the effects of S‐220 on all key CNS cell populations, as it included neurons, oligodendrocytes, astrocytes, microglia, and oligodendrocyte precursor cells. The results show that following S‐220 treatment, Epac2 activation conferred neuroprotection to neurons and oligodendrocytes following the in vitro injury. It also produced a permissive postinjury environment by reducing astrogliosis and microgliosis, which resulted in increased axonal outgrowth into the injury gap. Our data therefore suggest that elevating Epac2 is a novel repair strategy for CNS injury.

## Graphical abstract

**Study design.** An in vitro cortical mechanical injury model for central nervous system repair, which contained five major neural tissue cells, is established from rats. After 7 days in vitro, the injuries are introduced, and Epac2 agonist S‐220 treatments are applied immediately after injury. The results suggest that Epac2 activation is a novel repair strategy for CNS repair.

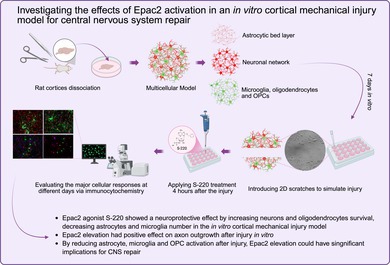

**Abbreviations**
AKT: Protein kinase B
AWERB: Animal Welfare and Ethics Review Board
BAD: Bcl‐2 antagonist of cell death
BNDF: Brain‐derived neurotrophic factor
cAMP: Cyclic adenosine 3′,5′‐monophosphate
CNS: Central nervous system
CREB: cAMP responsive element binding protein
CRI: Cell roundness index
CSPG: Chondroitin sulfate proteoglycan
DIV: Days in vitro
DPI: Days post injury
Epac: Exchange protein directly activated by cyclic adenosine 3′,5′‐monophosphate
ERK: Extracellular signal‐regulated kinase
GEF: Guanine nucleotide exchange factor
GFAP: Glial fibrillary acidic protein
Iba1: Ionized calcium binding adaptor molecule 1
iNOS: Inducible nitric oxide synthase
MBP: Myelin basic protein
mTOR: Mammalian target of rapamycin
NG2: Neuronal glial antigen 2
OPC: Oligodendrocyte precursor cell
PTPσ.: Protein tyrosine phosphatase sigma
RhoA: Ras‐homologous A
RT‐qPCR: Reverse transcription‐quantitative polymerase chain reaction
SCI: Spinal cord injury
SEM: Standard error of the mean
STAT3: Signal transducers and activators of transcription 3

## 1 Introduction

Millions of patients worldwide every year experience spinal cord injuries (SCIs) (Safdarian et al. 2023). This causes loss of neural function and has no cure, creating substantial social and economic challenges. Several factors impede SCI repair, including the limited intrinsic regenerative capacity of adult mammalian central nervous system (CNS) neurons, the formation of cavities and glial scars, and the presence of inhibitory molecules at the injury site.

Epac (exchange protein directly activated by cyclic adenosine 3′,5′‐monophosphate) has been associated with a broad spectrum of cellular functions that might be beneficial for the restoration of neural function, encompassing cell growth, adhesion, differentiation, division, exocytosis, and inflammation. Furthermore, it participates in regulating neurotransmission in both healthy and pathological states (Peace and Shewan 2011). Epac2 expression is predominantly confined to the CNS and the adrenal gland, with only limited presence in the heart, small intestine, and testis (Guijarro‐Belmar, Domanski, et al. 2021). Therefore, targeting Epac2 may offer a relatively neuron‐specific approach to augment intrinsic axonal growth and other processes beneficial to recovery from SCI (Murray and Shewan 2008). Activation of Epac2 reverses the postinjury inhibitory environment and promotes robust axon outgrowth in an ex vivo model of SCI (Guijarro‐Belmar, Viskontas, et al. 2019), which shows the beneficial and promising potential of Epac2 agonist therapy for SCI repair.

Evidence from that ex vivo SCI model suggests that Epac2 elevation by the agonist S‐220 can transform a post‐lesion inhibitory environment to one, which promotes axonal outgrowth (Guijarro‐Belmar, Viskontas, et al. 2019). However, the ex vivo model used in that study could not offer detailed and accurate assessment of responses of individual cell populations following injury. Moreover, it remains unclear if S‐220 conferred neuroprotection in the ex vivo model. In the present study, we use a relatively novel but simple in vitro model of CNS injury, which included neurons, oligodendrocytes, astrocytes, microglia, and oligodendrocyte precursor cells (OPCs) to examine the effects of S‐220 on all these key spinal cord cell populations. A scratch wound in this multicell co‐culture model replicates induction of various injury mechanisms that effectively mimic relevant pathologies relevant to CNS injury, including glial scarring, immune activation, and neuronal outgrowth (Wiseman 2023; Adams et al. 2024; Wiseman et al. 2025).

Our objectives were to assess the effects of S‐220 treatment after injury on these key cells and parameters, including neurons, oligodendrocytes, axonal outgrowth, astrocyte reaction, reactive microgliosis, and OPC infiltration from lesion edge. We propose that Epac2 activation by agonist S‐220 could offer a novel strategy in both neuroprotection and neuro‐regeneration for CNS injury repair.

## 2 Materials and Methods

### 2.1 Production of Mixed Cortical Cell Population: In Vitro 2D Multicell‐Type Cortical Injury Modeling

All procedures involving the use of live animals and animal tissues were performed in accordance with the UK Home Office (Scientific Procedures) Act, 1986 and were approved by the Animal Welfare and Ethics Review Board (AWERB) of the University of Aberdeen.

Cortices of Sprague Dawley rats of either sex at postnatal day 1 were harvested as a source for culturing cells. The tissue was dissociated enzymatically with 1 mL of Trypsin–EDTA (T4174, Sigma, UK) and 500 μL of DNase (DN25‐100MG, Merck, UK) and was shaken at 150 rpm at 37 °C (Labnet 211D Stainless Steel Incubator, Labnet, UK) for 20–30 min. The reaction was stopped by using 2 mL of FBS (Gibco Life Technologies, UK), along with 2 mL of Neurobasal media (Thermo‐Fisher scientific, UK) supplemented with B‐27 Supplement (50×, 2%, Gibco, UK), Glutamax (1%; Thermo‐Fisher scientific, UK), and Penicillin–Streptomycin (1%; Sigma, UK). The cells were plated at 1 x 10^6^ cells/mL, 300 μL per well on pre‐prepared PDL and laminin coated round 13‐mm glass coverslips in a 24‐well plate and incubated in a 37 °C, 5% CO_2_, 95% humidity environment. After 24 h after culturing, all media were removed, then 500 μL of complete Neurobasal media was added. During the culture, 50% of culture media was changed out every 48 h.

Once cultures had reached 90%+ confluency, around 7 days in vitro (DIV), a scratch was introduced down the center of the well (Figure 2.2 and 2.3). Using a sterile P200 pipette tip at a 90° angle to the well bottom, the tip was moved smoothly from the top of the well to the bottom in one continuous movement, keeping an even pressure throughout. The tip removed all cells in its path, creating a lesion area the width of the tip (400 μm). The cultures were left for 3 days post injury (DPI) or 7 DPI to allow cells in the lesion area to respond.

### 2.2 S‐220 Treatments

S‐220 (B 046, BioLog, UK) was used according to the manufacturer's instructions for Epac2 activation. For S‐220 treatment groups, 5 μM S‐220 was applied to the complete Neurobasal medium 4 h after injury. During the cultures, 50% medium was changed regularly every 48 h and fresh S‐220 was included as well.

### 2.3 Immunocytochemistry

Cells and explants were fixed with 4% PFA in PBS for 30 min, followed by incubation with 10% normal goat serum for 1 h, all at room temperature. Cells were then incubated with primary antibodies overnight at 4°C. We used the following primary antibodies: mouse anti‐β‐tubulin III (1:1000, T8578, Sigma‐Aldrich, UK), rabbit anti‐glial fibrillary acidic protein (GFAP) (1:500, AB5804, Millipore, UK), rabbit anti‐ionized calcium binding adaptor molecule 1 (Iba1) (1:1000, 019‐19741, Wako, Japan), rat antimyelin basic protein (MBP) (1:200, aa82‐87, Bio‐Rad, UK), and rabbit anti–neuronal glial antigen 2 (NG2) (1:500, ZRB5320, ZooMAb, UK). Following three washes with PBS, cells/explants were incubated for 2 h at room temperature with the appropriate secondary antibodies, including Alexa Fluor 488 anti‐Mouse (1:400, AB_2534069, Invitrogen, UK), Alexa Fluor 594 anti‐Mouse (1:200, AB_2534073, Invitrogen, UK), Alexa Fluor 568 goat anti‐Rabbit (1:200, AB_143157, Invitrogen, UK), and Alexa Fluor 488 goat anti‐Rat (1:200, AB_2534074, Invitrogen, UK). Coverslips were mounted with PBS/glycerol (1:8 ratio) after counterstaining with Hoechst 33342 (2 μg/mL in PBS; Sigma, UK). All primary/secondary antibodies and goat serum were prepared with PBS containing 0.2% Triton X‐100 (Sigma, UK) and 0.1% sodium azide (Sigma, UK).

### 2.4 Randomization and Blinding

Cultures were randomized to treatment groups, with plate position balanced across conditions. Although the researcher was aware of allocation during injury and treatment, all imaging and quantitative analyses were conducted on anonymized, coded datasets with randomized acquisition order and the same standard image acquisition settings for all images. Unblinding occurred only after final statistical analysis had been performed.

### 2.5 Imaging

An Eclipse Ti‐E fluorescence microscope (Ti‐E, Nikon Instruments Europe B.V., UK) was used to collect all images. Images were taken using a Nikon‐DS‐U3 camera with 20X objective magnification (CFI S Plan Fluor ELWD 20XC, Nikon Instruments Europe B.V., UK) and NIS‐elements 5.1.1 software (Nikon Instruments Europe B.V., UK) then saved in ND2 format, with files containing all image information. Where applicable, fluorescence images were merged using Fiji/ImageJ2 (version 2.14.0/1.54f).

### 2.6 Quantification

The quantification of images was conducted using Fiji/ImageJ2 software. The assessment of postinjury pathology was performed on 3 DPI and 7 DPI. Each analysis included a minimum of three biological replicates, with five regions imaged per condition across three to five coverslips.

#### 2.6.1 Cell Population Assessment

The numbers of cells immuno‐positive for β‐tubulin III, GFAP, Iba1, NG2, or MBP were quantified from respective fluorescent micrographs. To do this, five regions per culture were randomly selected in different locations on coverslips using the DAPI‐only channel on the fluorescent microscope. Percentages of cells were calculated by counting the proportion of cell marker–positive cells compared with total nuclei within the field.

#### 2.6.2 Number of Neurons/Oligodendrocytes/Astrocytes/Microglia Quantification

β‐Tubulin III+ cells, MBP+ cells, GFAP+ cells, and Iba+ cells were counted to identify neurons, oligodendrocytes, astrocytes, and microglia numbers. Micrographs were viewed in ImageJ, and the Crosshair tool was used to count cell numbers, then export numbers to excel.

#### 2.6.3 Astrocyte Astrogliosis and Morphological Quantification

All images were converted into 8‐bit files, and a threshold was applied to select only the area with GFAP immunoreactivity in ImageJ. The intensity of staining was determined by the mean optical density value. The mean background fluorescence proximate to each cell was subtracted from the measured fluorescence intensity of the cell area to give a background‐subtracted fluorescence intensity.

Semi‐quantitative analysis was used to evaluate astrocytic morphology in peri‐lesion astrocytes (within 100 μm of the lesion edge). In this model, GFAP+ cells were classified as Type 1 (flat), which meant astrocytes without cell processes, or Type 1 (processed), which were astrocytes that presented multiple processes. The percentage of astrocyte morphology in both types was calculated for each field.

#### 2.6.4 Microglia Infiltration and Morphological Quantification

Microglial infiltration was evaluated by counting the number of Iba1+ cells within the lesion site. Microglial morphology was characterized by a cell roundness index (CRI) to determine the reactive state (Ryo et al. 2016; Fernández‐Arjona et al. 2017). Iba1 fluorescent 20X magnification micrographs were analyzed in ImageJ. The freehand drawing tool was used to draw around individual Iba1+ cells that were peri‐lesional and distal to the lesion, producing measurements of cell perimeter, area, and Feret diameter, which were subsequently entered into the cell roundness formulae. For the latter, a value of 1 denotes a complete circle; anything less is progressively less round. Averages were plotted for statistical analysis.

#### 2.6.5 Neuronal Outgrowth Quantification

Lesion sites were immuno‐stained for β‐tubulin III to identify neurite extension into the lesion gap and co‐stained with DAPI to assist with identification of the lesion edge. Micrographs were viewed in ImageJ and fiber sprouting at the lesion edge was measured by the freehand line drawing tool. Here, an axon length from the lesion edge to its distal endpoint was traced and length measured. Note: neurite extension was not in a uniform direction, so this measurement was not distance into the lesion. Averages were calculated per coverslip then per experiment.

#### 2.6.6 OPC Infiltration Quantification

OPC infiltration was evaluated by counting the number of NG2+ cells within the lesion site from 20X magnification micrographs at 3/7 DPI, and five lesional areas were analyzed per repeat. Averages were plotted for statistical comparison.

### 2.7 Statistical Analysis

Data are presented as mean ± standard error of the mean (SEM). Each experiment had three to four biological replicates (*N*), and each biological replicate has three to five technical replicates (*n*). Statistical analyses were performed using GraphPad Prism 9. The normality test Shapiro–Wilk was performed to check the normal distribution of the data and suitability of parametric tests; normality was met with all the data.

Statistical analyses were performed using two‐way ANOVA with Bonferroni's post hoc analysis for cell population distribution quantification. One‐way ANOVA with Tukey post hoc analysis was applied for quantification of the number of neurons, oligodendrocytes, astrocytes, and microglia. Two‐way ANOVA with Tukey post hoc analysis was applied for quantification of perilesional astrocyte morphologies. Unpaired *t* test analysis was used for quantifications of perilesional astrocyte reactivity, perilesional microglial cell number, perilesional microgliosis cell area, perilesional microglia cell roundedness index, perilesional axonal outgrowth, and perilesional OPC infiltration. Statistically significant differences are indicated at *p* < 0.05 (*), *p* < 0.01 (**), *p* < 0.001 (***).

## 3 Results

### 3.1 All Major Types of Neural Cells Were Present in the Model

**FIGURE 1:**
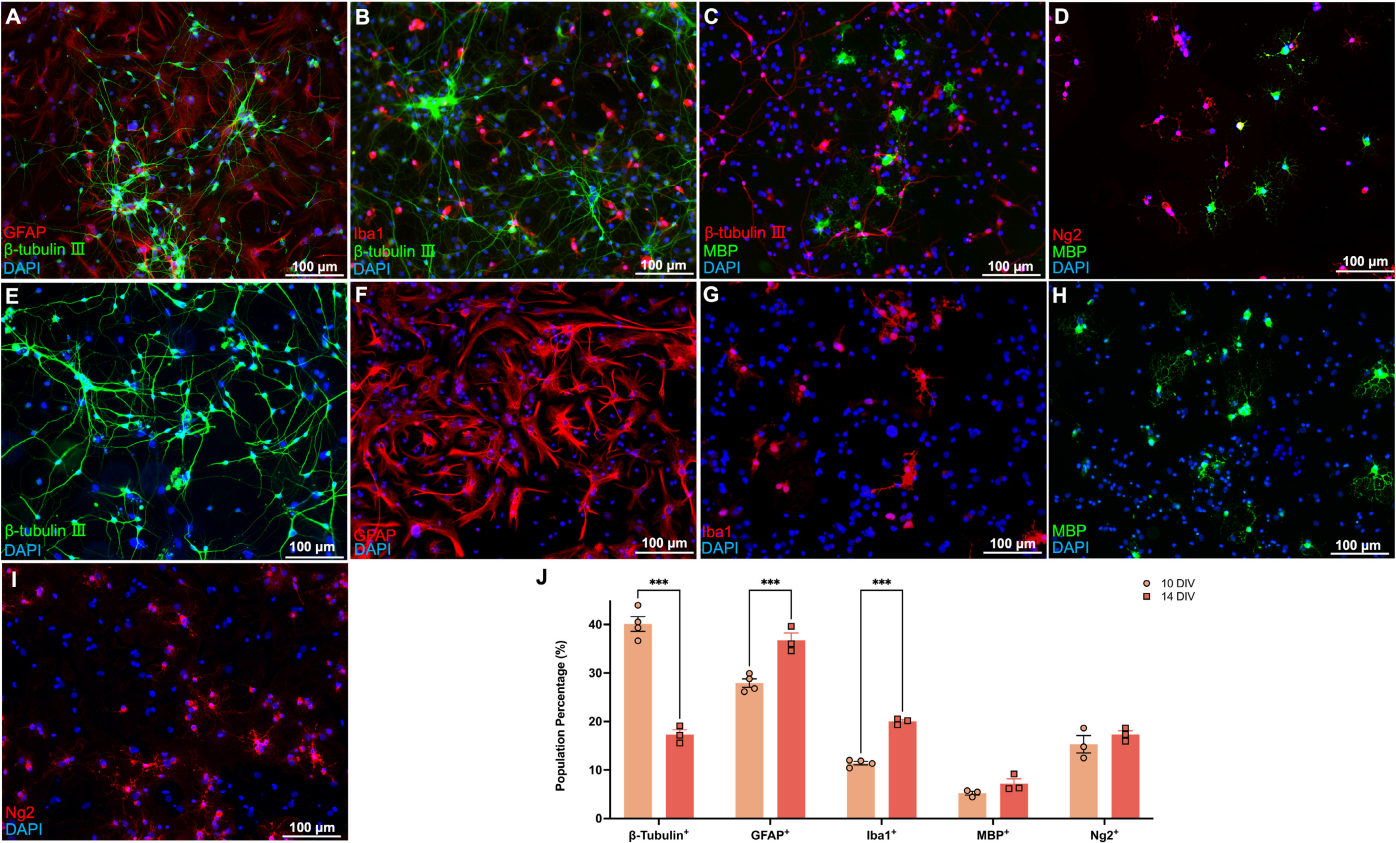
Multiple immunolabeling for GFAP, β‐tubulin III, Iba1, Ng2, and MBP demonstrated stratified relationships and characterization of population distribution. (A): Bed layer of astrocytes (GFAP+) was seen at the deepest focal plane; then a neuronal network (β‐tubulin III+) was found at the second field of focus. (B): Iba1+ microglia resting at the upper level of neurons. (C, D): MBP+ oligodendrocytes and Ng2+ OPCs at the top of culture. (E–I): Representative fluorescence micrographs of β‐tubulin III+ (neurons), GFAP+ (astrocytes), Iba1+ (microglia), MBP+ (oligodendrocytes), and NG2+ (OPCs). (J): The cells' population distribution. DAPI reveals nuclei in all graphs. Scale bar: 100 μm. Two‐way ANOVA with Bonferroni's post hoc analysis. *N* = 3–4. Data are expressed as mean ± SEM, *p* < 0.05 (*), *p* < 0.01 (**), and *p* < 0.001 (***).

Double immunolabeling revealed spatial relationships between astrocytes, neurons, microglia, oligodendrocytes, and OPCs in the cultures. With careful adjustment of the focal plane, it was clear there was a base layer of astrocytes (Figure 1A), then a dense neuronal network above the astrocytic layer (Figure 1A), above which were “resting” ramified microglia (Figure 1B) and oligodendrocytes/OPCs (Figure 1C) residing at the top of the culture.

To see if the different cellular populations were stable with time, individual CNS cell type abundance was compared between two different culturing times. Neurons were the most numerous cell type at 10 DIV, while astrocytes were the most numerous cell type at 14 DIV. The 14 DIV cultures showed a significant increase in the number of astrocytes compared to 10 DIV (14 DIV vs. 10 DIV: *p* < 0.001, two‐way ANOVA with Bonferroni's post hoc analysis, Figure 1J). There were significantly more microglia as well (14 DIV vs. 10 DIV: *p* < 0.001, two‐way ANOVA with Bonferroni's post hoc analysis, Figure 1J), with a substantial reduction in neuronal numbers (14 DIV vs. 10 DIV: *p* < 0.001, two‐way ANOVA with Bonferroni's post hoc analysis, Figure 1J). Unlike all the other cell types, the abundance of OPCs and oligodendrocytes did not significantly differ at 10 DIV and 14 DIV (Figure 1J).

### 3.2 Facile Induction of Injuries Is Feasible in the Model

To test the effects of injury, cultures were grown to 7 DIV, and penetrating injuries were introduced, then the corresponding quantification was performed relative to the resulting lesion at 3 DPI and 7 DPI, respectively.

**FIGURE 2:**
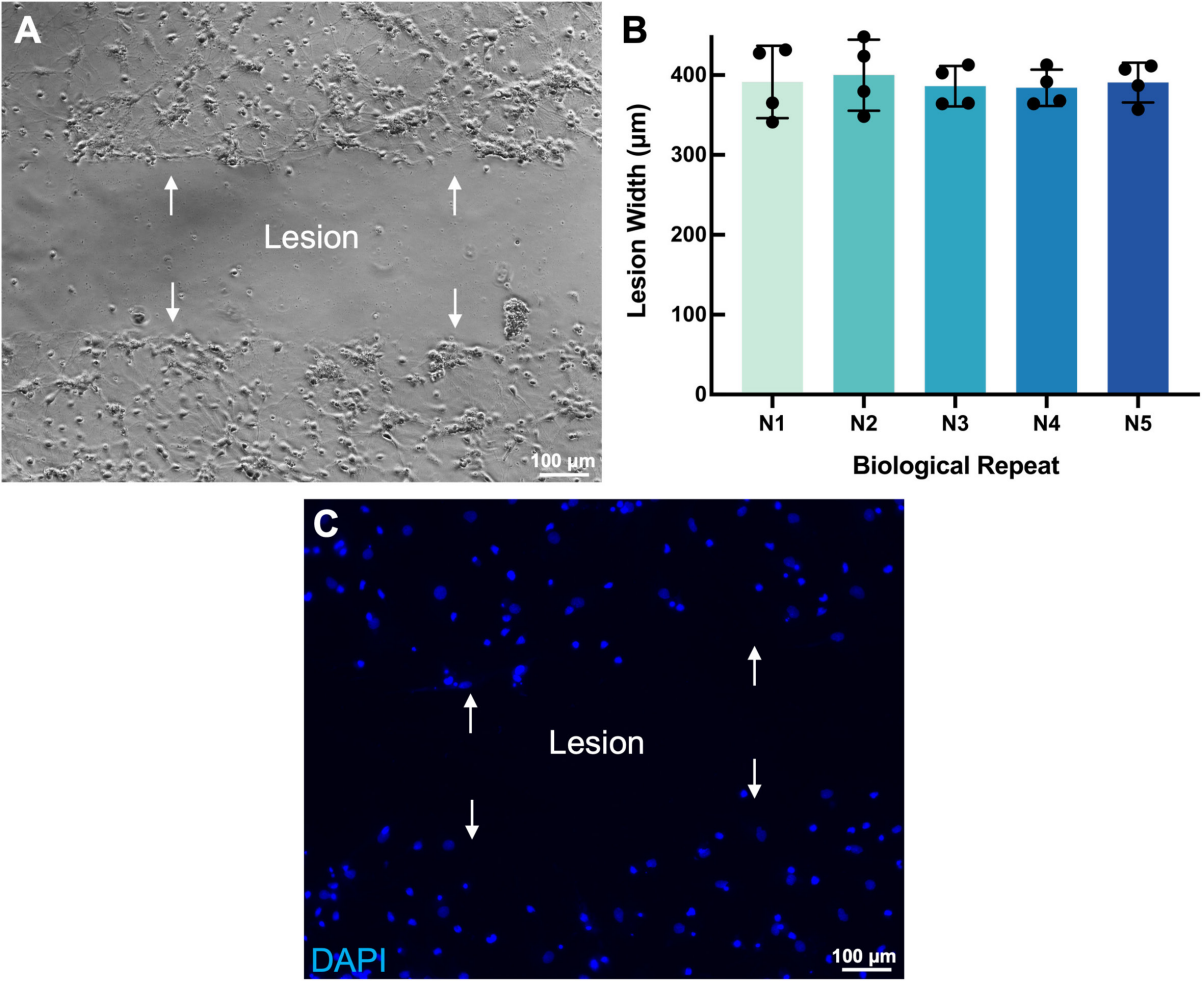
Reproducibility of injury width. (A): The brightfield micrograph demonstrating the defined injury margins with minimal intralesional debris immediately after post injury, white arrows showed the injury border. (B): Representative fluorescent micrograph demonstrating a defined injury margin can be distinguished under the nuclear immunolabel DAPI, white arrows showed the injury border. (C): Bar chart showing consistently reproducible lesion widths across different biological replicates. Scale bar: 100 μm. *N* = 5. Data are expressed as mean ± SEM.

Injury lesions introduced into the cultures at 7 DIV are shown in Figure 2. The tip removed all cells in its path, creating a lesion area the width of the tip (400 μm), and the lesions produced were consistent and reproducible in width over all cultures (Figure 2B). The DAPI nuclear stain indicated defined injury margins at 3 DPI, but with few cellular migrations into the lesion area (Figure 2C).

### 3.3 S‐220 Treatment Confers Neuroprotection

For testing neuroprotective potential, the number of neurons, oligodendrocytes, astrocytes, and microglia was quantified. The cells in the injured and S‐220 groups were counted around the lesion border, which was within 0–100 μm away from the lesion edge.

#### 3.3.1 S‐220 Treatment Increases Neuron Numbers and Promotes Neurons Survival

**FIGURE 3:**
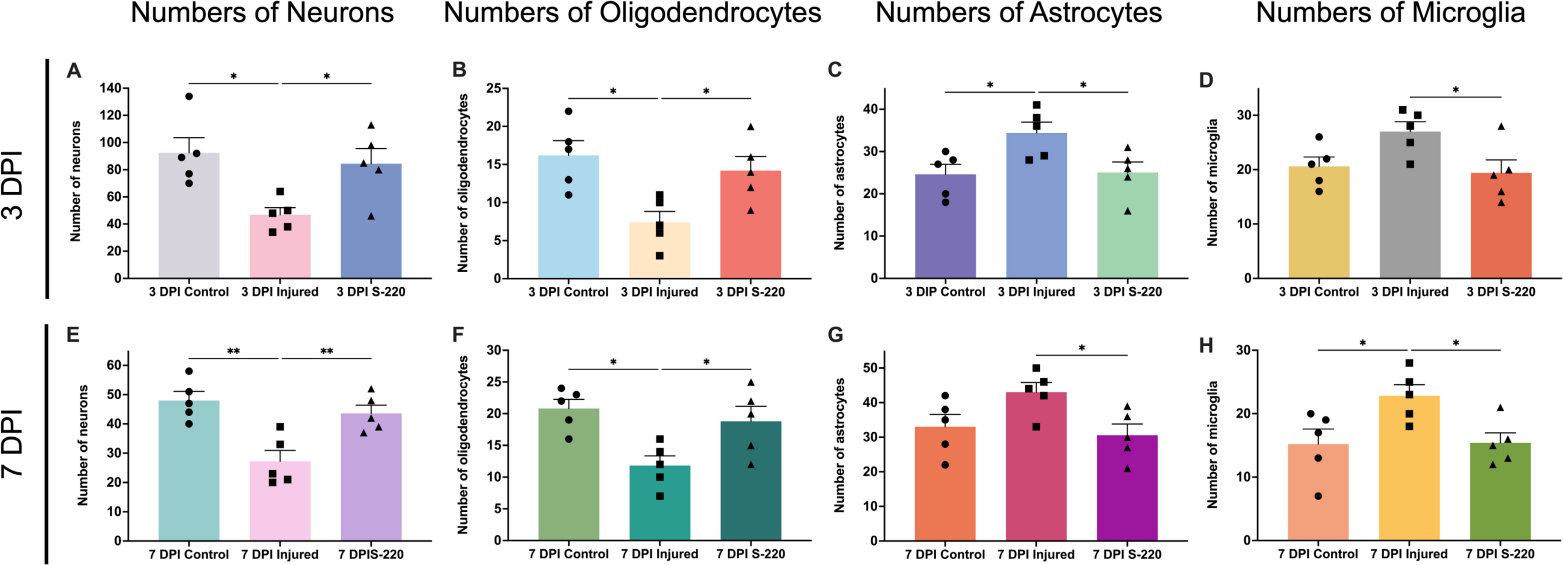
Quantification of cell numbers at 3 DPI and 7 DPI. (A, E): Number of neurons at 3 DPI and 7 DPI, S‐220 treatment significantly increased the number of neurons when compared with the injured group. (B, F): Number of oligodendrocytes at 3 DPI and 7 DPI, S‐220 treatment significantly increased the number of oligodendrocytes when compared with the injured group. (C, G): Number of astrocytes at 3 DPI and 7 DPI, S‐220 treatment significantly decreased the number of astrocytes when compared with the injured group. (D, H): Number of microglia at 3 DPI and 7 DPI, S‐220 significantly decreased the number of microglia compared to the injured group. One‐way ANOVA with Tukey post hoc analysis. *N* = 5. Data are expressed as mean ± SEM, *p* < 0.05 (*), *p* < 0.01 (**), *p* < 0.001 (***).

After injury, the number of neurons decreased compared to the noninjury control (3 DPI injured vs. 3 DPI control: *p* = 0.015, one‐way ANOVA with Tukey post hoc analysis, Figure 3A) (7 DPI injured vs. 7 DPI control: *p* = 0.002, one‐way ANOVA with Tukey post hoc analysis, Figure 3E). Following treatment, S‐220 significantly increased the number of neurons compared to the injury only groups (3 DPI S‐220 vs. 3 DPI injured: *p* = 0.042, one‐way ANOVA with Tukey post hoc analysis, Figure 3A) (7 DPI S‐220 vs. 7 DPI injured: *p* = 0.01, one‐way ANOVA with Tukey post hoc analysis, Figure 3E). Importantly, there was no significant difference between S‐220 treatment groups and noninjury control groups (3 DPI S‐220 vs. 3 DPI control: *p* = 0.828, one‐way ANOVA with Tukey post hoc analysis, Figure 3A) (7 DPI S‐220 vs. 7 DPI control: *p* = 0.614, one‐way ANOVA with Tukey post hoc analysis, Figure 3E).

#### 3.3.2 S‐220 Treatment Increased Oligodendrocyte Number

Similar benefits were seen with oligodendrocyte numbers. First, the oligodendrocytes cell count was decreased after injury when compared with noninjury control (3 DPI injured vs. 3 DPI control: *p* = 0.01, one‐way ANOVA with Tukey post hoc analysis, Figure 3B) (7 DPI injured vs. 7 DPI control: *p* = 0.012, one‐way ANOVA with Tukey post hoc analysis, Figure 3F). Conversely, after S‐220 treatment, the oligodendrocyte numbers were significantly increased (3 DPI S‐220 vs. 3 DPI injured: *p* = 0.044, one‐way ANOVA with Tukey post hoc analysis, Figure 3B) (7 DPI S‐220 vs. 7 DPI injured: *p* = 0.048, one‐way ANOVA with Tukey post hoc analysis, Figure 3F). Indeed, again, there was no significant difference between S‐220 treatment groups and noninjury control groups (3 DPI S‐220 vs. 3 DPI control: *p* = 0.707, one‐way ANOVA with Tukey post hoc analysis, Figure 3B) (7 DPI S‐220 vs. 7 DPI control: *p* = 0.728, one‐way ANOVA with Tukey post hoc analysis, Figure 3F).

#### 3.3.3 S‐220 Treatment Reduced Astrocyte Number and Reactive Astrogliosis

The astrocyte numbers were significantly increased at 3 DPI when compared to the noninjury control (3 DPI injured vs. 3 DPI control: *p* = 0.04, one‐way ANOVA with Tukey post hoc analysis, Figure 3C). There was also a trend of increasing astrocytes' number at 7 DPI when compared with the control group (Figure 3G). Comparing S220 treatment groups with the injured groups, the numbers of astrocytes were significantly reduced at both 3 DPI (3 DPI S‐220 vs. 3 DPI injured: *p* = 0.049, one‐way ANOVA with Tukey post hoc analysis, Figure 3C) and 7 DPI (7 DPI S‐220 vs. 7 DPI injured: *p* = 0.045, one‐way ANOVA with Tukey post hoc analysis, Figure 3G).

**FIGURE 4:**
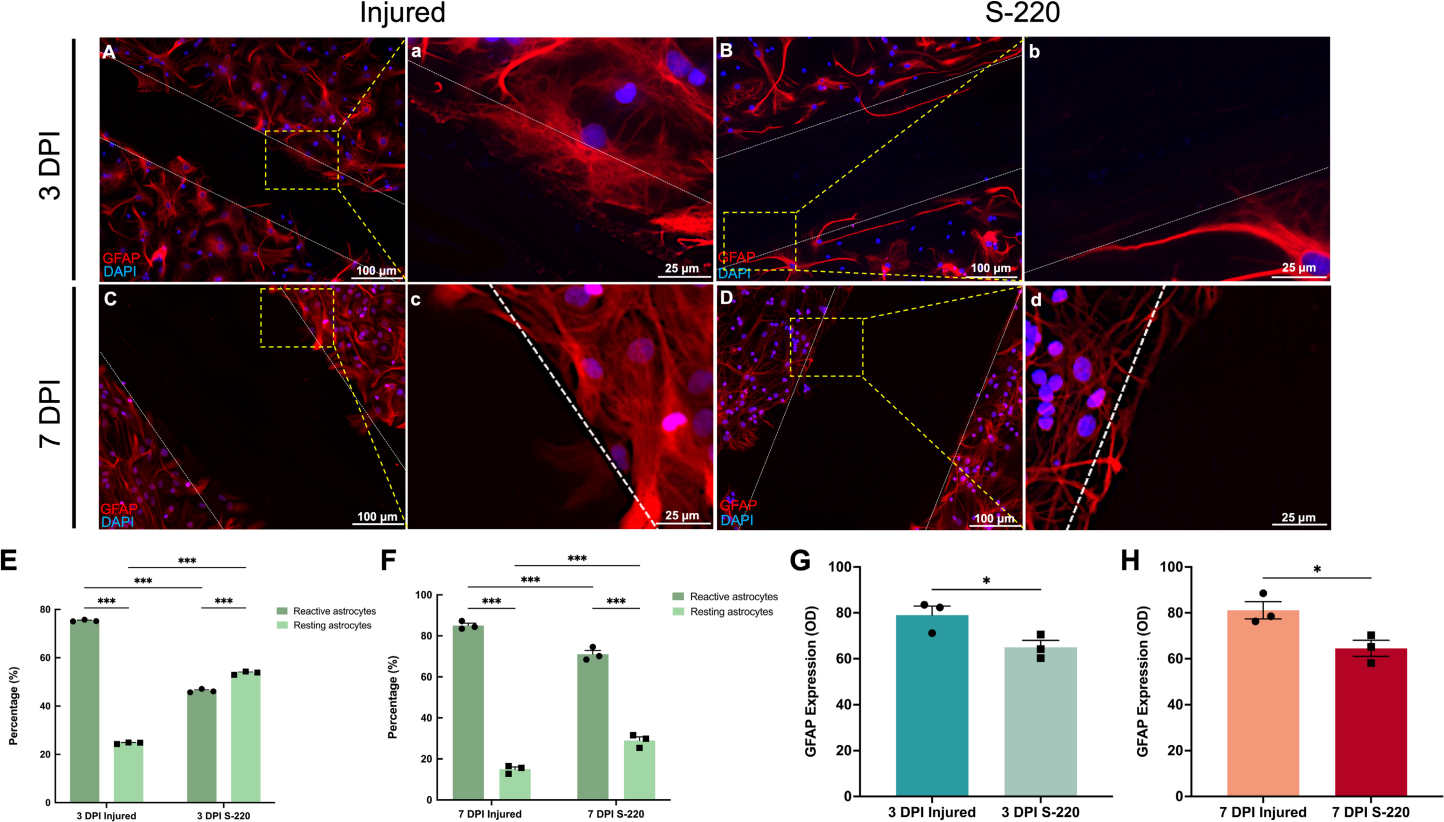
Characterization and quantification of perilesional astrocytes morphologies at 3 DPI and 7 DPI. (A, C): Lesional infiltration of astrocytes at 3 DPI and 7 DPI, showing astrocytic ruffles of injury‐activated astrocytes beyond the injury border, forming a glial scar of reactive astrocytes. White dashed lines indicated injury margins. The yellow dotted box indicated that this part was zoomed in to obtain a, c. (B, D): S‐220 treated perilesional astrocytes at 3 DPI and 7 DPI, showing astrocytes around lesion border presented the fibrous morphology after S‐220 treatment. White dashed lines indicated injury margins. The yellow dotted box indicated that this part was zoomed in to obtain b, d. (E, F): Quantitative analysis of morphology distribution percentage for perilesional astrocytes at 3 DPI and 7 DPI. (G, H): Quantification of GFAP reactivity intensity at 3 DPI and 7 DPI. The S‐220 treatment led to a significant decrease in GFAP immunoreactivity in perilesional astrocytes. Scale bar: A, B, C, D 100 μm; a, b, c, d 25 μm. E, F: Two‐way ANOVA with Tukey post hoc analysis. G, H: Unpaired *t* test. *N* = 3. Data are expressed as mean ± SEM, *p* < 0.05 (*), *p* < 0.01 (**) and *p* < 0.001 (***).

To define if this model demonstrated injury‐induced astrogliosis, astrocyte morphology and GFAP expression were further evaluated at the injury site. Reactive astrocytes commonly show a morphology of being hypertrophic and/or extending ruffles, while resting astrocytes are star‐shaped and radially project a multitude of repeatedly branching variable diameter cellular processes (Cheng et al. 2019). Astrocytes were defined as perilesional or proximal astrocytes if they were 0–100 μm away from the lesion edge. Inspection of the immunofluorescence images showed astrocytic ruffles of injury‐activated astrocytes beyond the injury border. A glial scar of fibrous astrocytes with enhanced GFAP expression was also observed at both 3 DPI and 7 DPI (Figure 4A, C).

S‐220 treatment significantly reduced the proportion of reactive astrocytes (Reactive astrocytes: 3 DPI S‐220 vs. 3 DPI injured: *p* < 0.001, two‐way ANOVA with Tukey post hoc analysis, Figure 4E) (reactive astrocytes: 7 DPI S‐220 vs. 7 DPI injured: *p* < 0.001, two‐way ANOVA with Tukey post hoc analysis, Figure 4F). Instead, it seemed rather that the astrocytes changed into resting states, as the percentages of astrocytes, which presented fibrous phenotypes were significantly raised (resting astrocytes: 3 DPI S‐220 vs. 3 DPI injured: *p* < 0.001, two‐way ANOVA with Tukey post hoc analysis, Figure 4E) (resting astrocytes: 7 DPI S‐220 vs. 7 DPI injured: *p* < 0.001, two‐way ANOVA with Tukey post hoc analysis, Figure 4F).

Finally, GFAP immunoreactivity was significantly less after S‐220 treatment at both 3 DPI and 7 DPI (3 DPI S‐220 vs. 3 DPI injured: *p* = 0.047, unpaired *t* test, Figure 4G) (7 DPI S‐220 vs. 7 DPI injured: *p* = 0.032, unpaired *t* test, Figure 4H).

#### 3.3.4 S‐220 Treatment Decreased Microglia Number and Attenuated Reactive Microgliosis Infiltration

The number of microglia at 3 DPI showed an increasing trend when compared to the noninjury control group (Figure 3D), and this increase was significant at 7 DPI compared with the control (7 DPI injured vs. 7 DPI control: *p* = 0.041, one‐way ANOVA with Tukey post hoc analysis, Figure 3H). After S‐220 treatments, compared to the injury‐only groups, the numbers of microglia decreased significantly at both 3 DPI (3 DPI S‐220 vs. 3 DPI injured: *p* = 0.049, one‐way ANOVA with Tukey post hoc analysis, Figure 3D) and 7 DPI (7 DPI S‐220 vs. 7 DPI injured: *p* = 0.047, one‐way ANOVA with Tukey post hoc analysis, Figure 3H).

**FIGURE 5:**
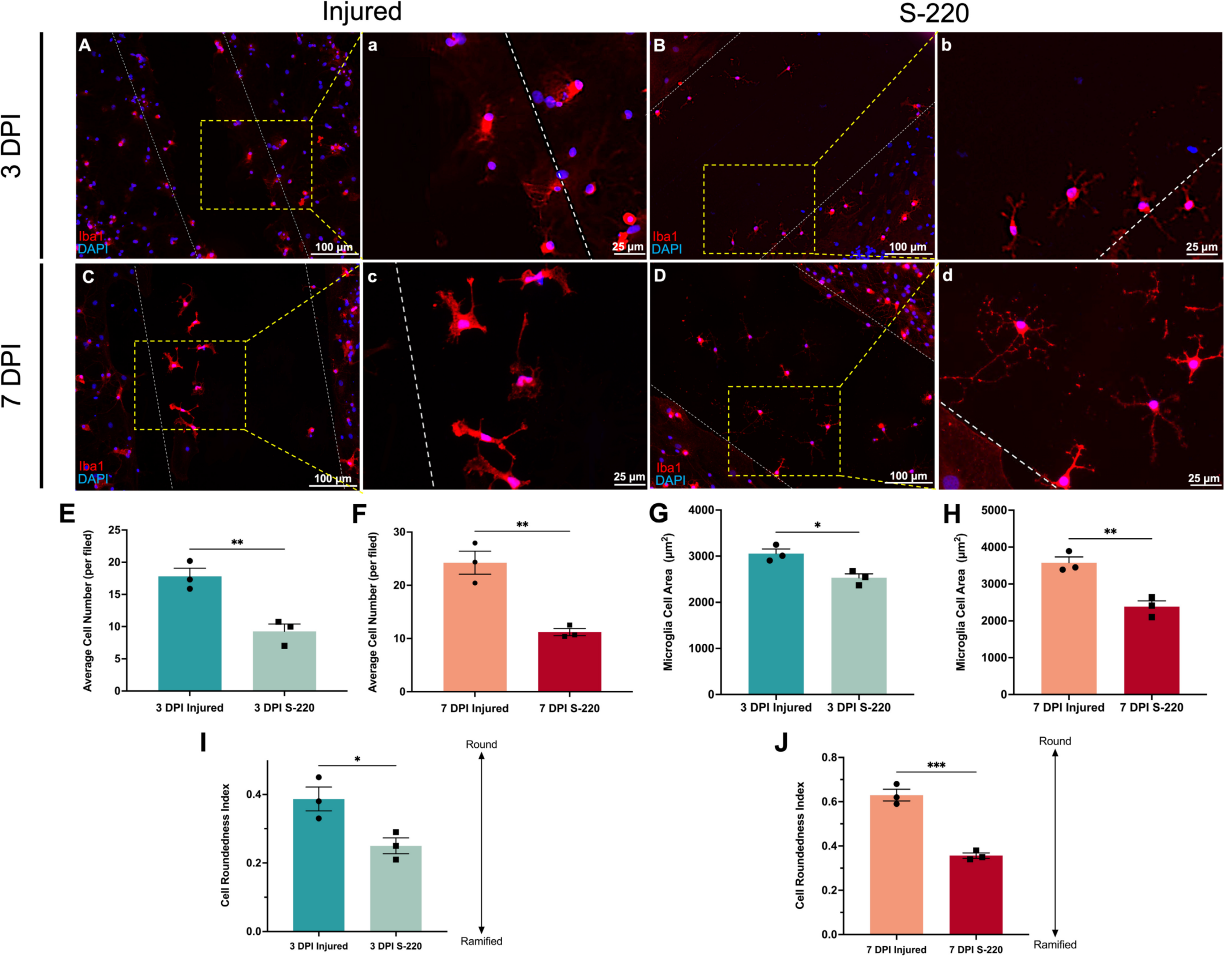
Characterization and quantification of morphologies and reactivity of perilesional microglia at 3 DPI and 7 DPI. (A, C): Lesional infiltration of Iba1+ microglia at 3 DPI and 7 DPI, white dashed lines indicated injury margins. The yellow dotted box indicated that this part was zoomed in to obtain a, e. (B, D): Perilesional microglia in S‐220 treatment groups at 3 DPI and 7 DPI, showing microglia infiltration in the lesion were significantly decreased after the S‐220 treatment. White dashed lines indicated injury margins. The yellow dotted box indicated that this part was zoomed in to obtain b, f. (E, F): Quantitative analysis of injury‐induced microglial lesion infiltration at 3 DPI and 7 DPI. (G, H): Quantitative analysis of microglial cell area at 3 DPI and 7 DPI. (I, J): Quantitative analysis of microglial cell round index. Scale bar: A, B, C, D, E, F, G, H 100 μm; a, b, c, d, e, f, g, h 25 μm. Unpaired *t* test. N = 3. Data are expressed as mean ± SEM, *p* < 0.05 (*), *p* < 0.01 (**) and *p* < 0.001 (***).

Like the astrocytes, perilesional microglia were defined as microglia, which were 0–100 μm away from the lesion edge. Iba1+ microglia infiltrated the lesion (Figure 5A, C) and exhibited distinct activated hypertrophic profiles (Figure 5a, c). After the S‐220 treatment, microglia infiltration in the lesion was significantly less (3 DPI S‐220 vs. 3 DPI injured: *p* = 0.008, unpaired *t* test, Figure 5E) (7 DPI S‐220 vs. 7 DPI injured: *p* = 0.005, unpaired *t* test, Figure 5F).

In addition, S‐220 treatment also significantly decreased the perilesional microglia cell area at 3 DPI and 7 DPI (3 DPI S‐220 vs. 3 DPI injured: *p* = 0.018, unpaired *t* test, Figure 5G) (7 DPI S‐220 vs. 7 DPI injured: *p* = 0.006, unpaired *t* test, Figure 5H).

Finally, microglial morphology was characterized by CRI. S‐220 treatment significantly decreased the CRI of microglia within the lesion site in cultures (3 DPI S‐220 vs. 3 DPI injured: *p* = 0.031, unpaired *t* test, Figure 5I) (7 DPI S‐220 vs. 7 DPI injured: *p* < 0.001, unpaired *t* test, Figure 5J).

### 3.4 S‐220 Treatment Results in Increased Lesional Axonal Profiles

**FIGURE 6:**
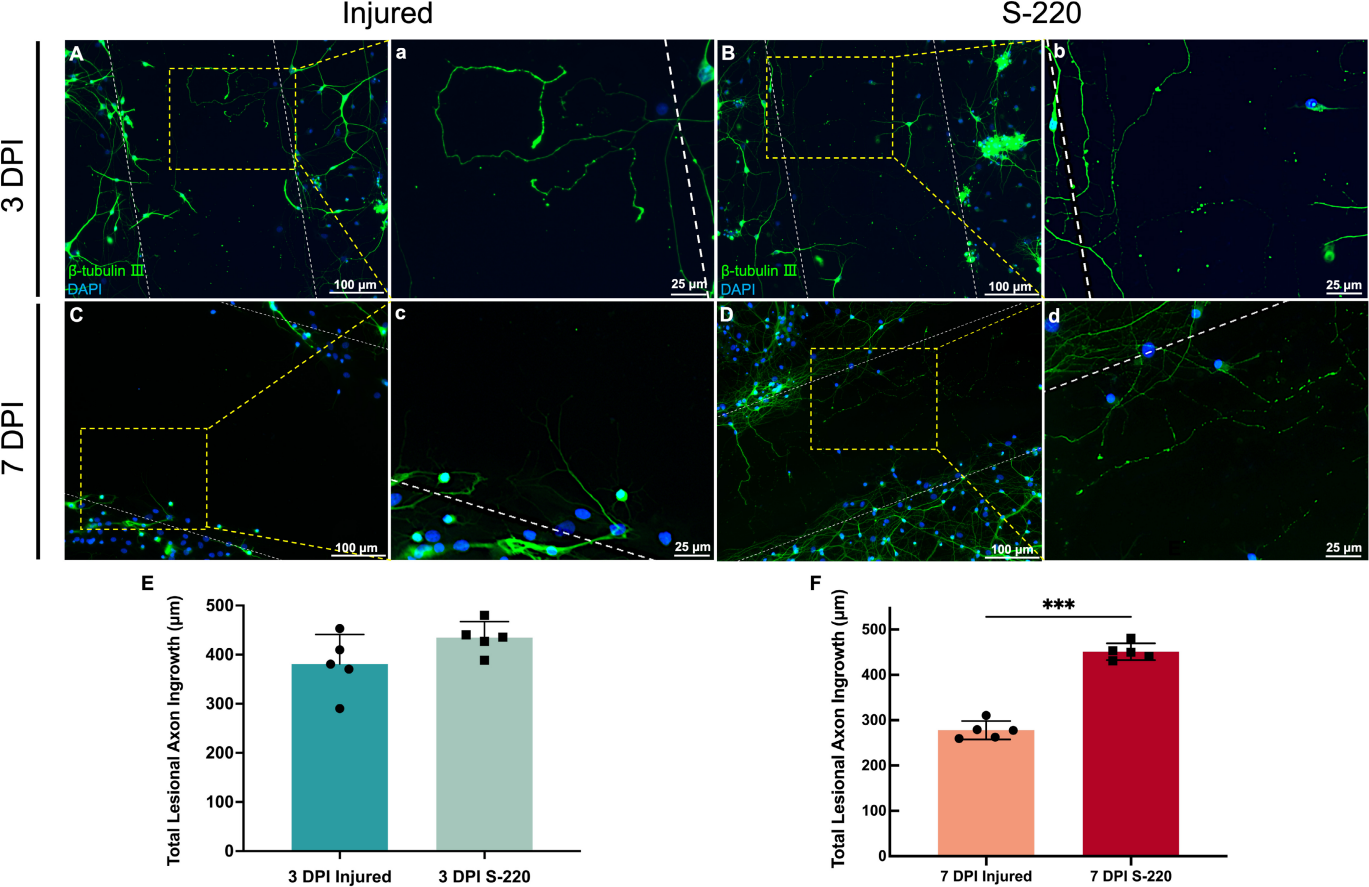
Representative micrograph and quantification of the substantial β‐tubulin III+ axonal outgrowth from the lesion edge at 3 DPI and 7 DPI. (A, C): Axon outgrowth at 3 DPI and 7 DPI, white dashed lines indicated injury margins. The yellow dotted box indicated that this part was zoomed in to obtain a, c. (B, D): Axon outgrowth with S‐220 at 3 DPI and 7 DPI, white dashed lines indicated injury margins. The yellow dotted box indicated that this part was zoomed in to obtain b, d. (E): Quantification of axonal outgrowth with or without S‐220 treatment after lesion at 3 DPI. (F): Quantification of axonal outgrowth with or without S‐220 treatment after lesion at 7 DPI, S‐220 treatment significantly promoted the axonal outgrowth in the injury site. Scale bar: A, B, C, D, 100 μm; a, b, c, d, 25 μm. Unpaired *t* test. N = 5. Data are expressed as mean ± SEM, *p* < 0.05 (*), *p* < 0.01 (**) and *p* < 0.001 (***).

When axons were severed at the injury edges and the response quantified, substantial axonal outgrowth from the lesion edge into the lesion gap was observed by 3 DPI (Figure 6A). Following S‐220 treatment, neurite outgrowth in the gap was significantly greater than untreated injured‐only groups at 3 DPI (3 DPI S‐220 vs. 3 DPI injured: *p* = 0.118, unpaired *t* test, Figure 6B, E). Substantial axonal outgrowth was observed from the lesion edge by 7 DPI in controls (Figure 6C). Nevertheless, after applying S‐220 treatment, for 7 DPI, the outgrowth was significantly enhanced by almost 1.7‐fold after the treatment of S‐220 (7 DPI S‐220 vs. 7 DPI injured: *p* < 0.001, unpaired *t* test, Figure 6F).

### 3.5 S‐220 Attenuates OPC Infiltration Within the Injury Site

**FIGURE 7:**
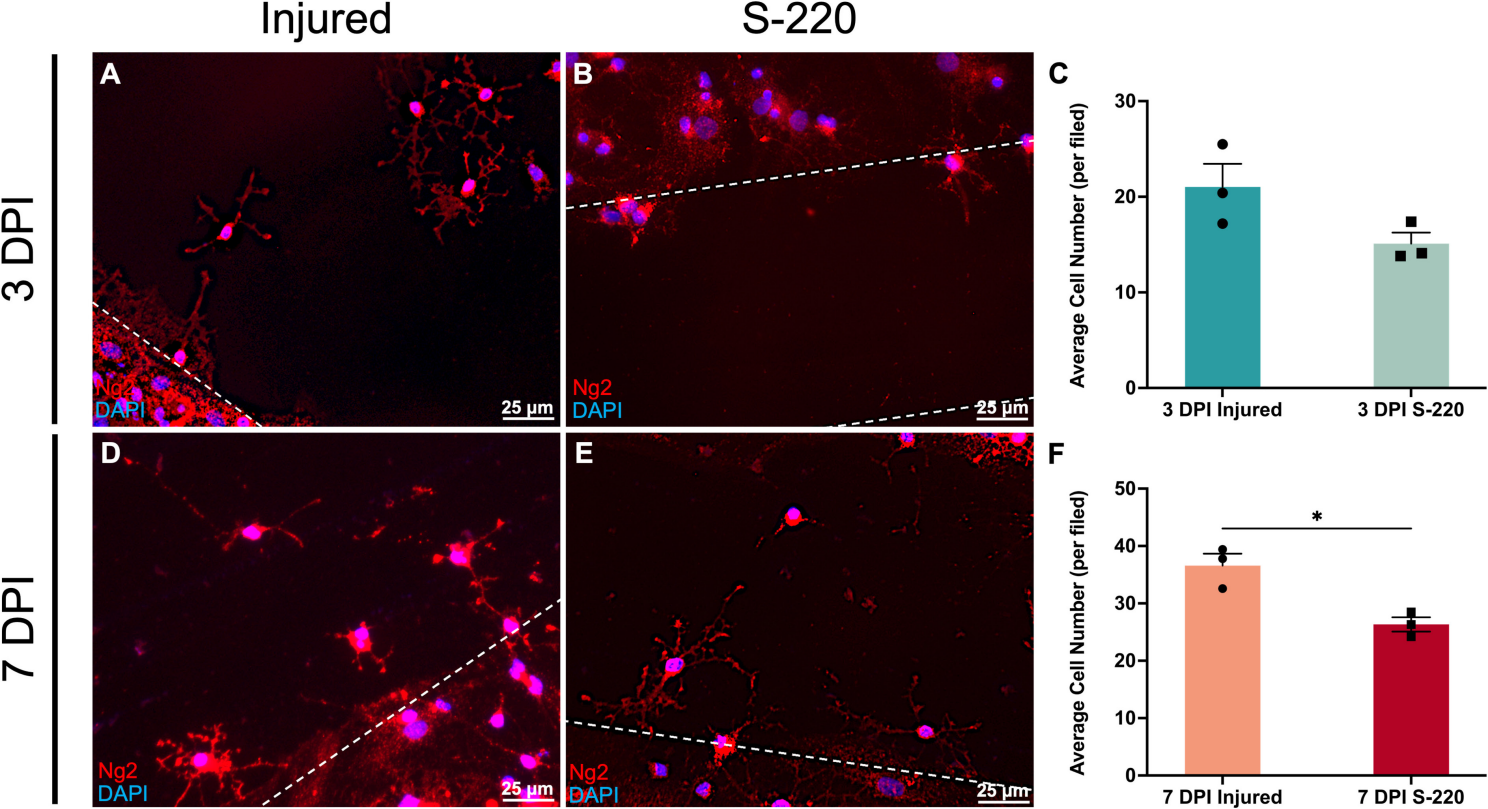
Characterization of OPCs morphologies and infiltration in lesion site at 3 DPI and 7 DPI. (A, D): OPCs infiltration into the lesion area at 3 DPI and 7 DPI, the white dashed lines indicated injury margins. (B, E): Perilesional OPCs in S‐220 treatment groups at 3 DPI and 7 DPI, the white dashed lines indicated injury margins. (C): Quantitative analysis of average OPC number in lesion site at 7 DPI. (F), Quantitative analysis of average OPC number in lesion site, the OPC number in lesion site were significantly decreased after S‐220 treatment at 7 DPI. Unpaired *t* test. Scale bar: 25 μm. N = 3. Data are expressed as mean ± SEM, *p* < 0.05 (*), *p* < 0.01 (**) and *p* < 0.001 (***).

As well as axons, NG2+ OPCs also infiltrated the lesion site post injury without treatment (Figure 7A, D). S‐220 treatment had no significant effect on the OPC number in the lesion site at 3 DPI (Figure 7C). However, the S‐220 treatment attenuated OPC infiltration within the injury site at 7 DPI, with the OPC number within the lesion site being significantly decreased (7 DPI S‐220 vs. 7 DPI injured: *p* = 0.013, unpaired *t* test, Figure 7F). For both injured OPCs and treated OPCs, there was no evidence of OPC nuclear doublets within the lesion, which would have revealed proliferation into the lesion site, indicating they were not proliferating here.

## 4 Discussion

In this study, we found that the Epac2 activation had an important beneficial modulatory role during repair in a novel in vitro multicellular CNS injury model. Our data also demonstrated for the first time that the Epac2 agonist S‐220 conferred neuroprotective effects. S‐220 treatment immediately after injury significantly increased the survival of neurons and oligodendrocytes. In addition, S‐220 treatment significantly decreased astrogliosis and microgliosis compared to the nontreated injured groups. Finally, it also significantly promoted lesional axonal outgrowth either directly or because of neuroprotection.

Epac2, which functions downstream of cAMP, is predominantly expressed in postnatal neural tissue, representing a relevant target and pertinent candidate for facilitating SCI repair (Guijarro‐Belmar, Domanski, et al. 2021). Epac2 activates many repair‐relevant mechanisms (Qiu et al. 2000; Enserink et al. 2002; Mei et al. 2002; Wang et al. 2006; Nijholt et al. 2008). The protein kinase B (AKT) pathway is crucial for cell survival, as it phosphorylates and inactivates components of the apoptotic machinery, such as Bcl‐2 antagonist of cell death (BAD) and caspase‐9, thereby promoting neuronal survival (Rai et al. 2019). In murine primary cortical neurons, Epac2 activation enhances phosphorylation of AKT, supporting neuronal survival and memory processes (O'Donovan et al. 2014). Activation of Epac2 is associated with the modulation of p38 MAPK activity, contributing to increased neuronal survival under stress conditions (Emery et al. 2014). Activation of extracellular signal‐regulated kinase (ERK) 1/2 leads to the upregulation of anti–apoptotic genes, such as BCL‐2 and BCL‐xL (Boucher et al. 2000), and suppresses caspase‐3 activity, reducing apoptosis (Oh et al. 2016). Studies have shown that sustained ERK activation protects against glutamate‐induced neuronal death (Almeida et al. 2005). Epac2 activation may also lead to the activation of cAMP responsive element binding protein (CREB), which then enhances the expression of neuroprotective genes, such as brain‐derived neurotrophic factor (BDNF) (Wei et al. 2016). Because the differentiation, maturation, and myelination of oligodendrocytes are dependent on cAMP‐mediated ERK/CREB and p38 MAPK/CREB pathways (Bhat et al. 2007), it is highly likely that Epac2 activation in oligodendrocytes would help their survival via the upregulation of anti–apoptotic genes and neuroprotective genes seen with neuronal survival. All these effects would benefit CNS repair.

Reactive astrocytes and microglia can release pro‐inflammatory cytokines after CNS injury, contributing to further neuronal and oligodendrocyte death (Allan and Rothwell 2001; Guttenplan et al. 2020; Lawrence et al. 2023). The administration of general cAMP agonists can suppress glial differentiation and activation (Miller et al. 1994; Fiebich et al. 1997; Apicelli et al. 2003; Lee et al. 2009; Kimelberg 2010; Paco et al. 2016). Elevation of cAMP through nonselective agonists can lead to an increase in arginase I production, a reduction in nitrite release, and an adoption of resting morphology in microglial BV‐2 cells when co‐treated with TNF‐α or LPS (Steininger et al. 2011; Ghosh et al. 2016). Our previous work has shown that Epac2 activation by S‐220 reduces astrocyte and microglial activation by LPS in cultured astrocytes and microglia (Guijarro‐Belmar, Domanski, et al. 2021). Therefore, the Epac2 activation in the present study may have reduced proliferation and activation of astrocytes and microglia, thereby reducing pro‐inflammatory mediators produced by these cells and contributing to the survival of neurons and oligodendrocytes.

Epac2 plays a pivotal role in promoting neurite outgrowth in neurons through several intracellular signaling pathways. Epac2 acts as a guanine nucleotide exchange factor (GEF) for Rap1B, a small GTPase that plays a role in cytoskeletal dynamics (Urrutia and González‐Billault 2023). When Epac2 was activated by the specific agonist 8‐pCPT, there was an increase in the levels of GTP‐bound Rap1B in cortical neurons (Muñoz‐Llancao et al. 2015). This activation leads to the formation of multiple axons and promotes axonal elongation, demonstrating Epac2's importance for axonal development (Urrutia and González‐Billault 2023). In PC12 cells, a well‐established model for sympathetic neurons, overexpression of Epac2 leads to increased neurite outgrowth, a process that underscores the role of Epac2 in facilitating neurite extension through ERK pathway activation (Liu et al. 2008). Furthermore, Epac2 activation can counteract inhibitory signals in the extracellular environment. Our previous work found that S‐220 treatment promoted neurite outgrowth in cultured rat cortical neurons in the presence of chondroitin sulfate proteoglycans (CSPGs), which are known to inhibit axonal growth. This suggests that Epac2 activation may help neurons overcome extracellular inhibitory cues, facilitating neurite extension (Guijarro‐Belmar, Viskontas, et al. 2019). Collectively, these observations highlight the central role of Epac2 in promoting intrinsic neurite outgrowth by modulating the neuronal response to extracellular inhibitory factors. It is conceivable that increased survival of neurons through neuroprotective mechanisms mentioned above may have also contributed to the increased neurite outgrowth observed here.

Epac2 activation promotes neurite outgrowth not only through direct neuronal mechanisms but also by modulating the activity of astrocytes and microglia, thereby creating a more permissive environment for axonal regrowth after injury. Astrocytes become reactive after CNS injury, characterized by hypertrophy and upregulation of GFAP, contributing to the formation of a glial scar and production of CSPGs that impede axonal regrowth. Microglia, the resident immune cells of the CNS, also become activated following mechanical injury, releasing pro‐inflammatory mediators, which further contribute to the hostile environment for neuronal regeneration after injury. Activation of Epac2 using the specific agonist S‐220 has been shown to attenuate the astrocytes' reactive phenotype and mitigate microglial activation. Our previous in vitro studies demonstrated that S‐220 treatment reduced GFAP expression and maintained astrocytes in a nonreactive, process‐bearing morphology. In vitro experiments with LPS‐stimulated microglia demonstrate that treatment with S‐220 reduced the expression of inducible nitric oxide synthase (iNOS) and nitrite release, markers of microglial activation (Guijarro‐Belmar, Viskontas, et al. 2019). The previous study in an ex vivo model of SCI further revealed that S‐220–treated astrocytes adopt an elongated morphology resembling radial glial progenitors, which are known to guide axonal growth during development (Guijarro‐Belmar, Viskontas, et al. 2019). Meanwhile, the S‐220 treatment in the ex vivo model resulted in microglia exhibiting a resting morphology with decreased cell body size, indicative of reduced activation. The combined modulation of astrocyte and microglial activity by Epac2 activation leads to a transformation of the lesion environment from inhibitory to supportive of neurite outgrowth, even across lesion gaps (Guijarro‐Belmar, Viskontas, et al. 2019). This suggests that Epac2 activation can reprogram glial responses to injury, facilitating a regenerative milieu conducive to neurite extension.

While our model enabled simultaneous assessment of neurons, astrocytes, microglia, oligodendrocytes, and OPCs under controlled conditions, its cellular origin from the cortex imposes important considerations for specific translation to repair in other parts of the CNS, such as SCI translation. Cortical and spinal astrocytes show region‐specific phenotypes and reactivity set points, including higher baseline and injury‐related GFAP/IL‐6/signal transducers and activators of transcription 3 (STAT 3) levels in spinal cord astrocytes, indicating that gliosis and trophic signaling captured in cortical cultures may not map one‐to‐one onto spinal tissue (Yoon et al. 2017). Beyond this, astrocyte heterogeneity across cortex, cerebellum, and spinal cord is well documented and partly linked to NOTCH1‐dependent programs, further supporting responses to injury and pharmacology (Hu et al. 2019). Microglia also differ by CNS compartment; single‐cell analyses identify overlapping yet distinct cortical versus spinal microglial populations and plasticity states, implying that inflammatory trajectories after injury in cortical cultures may diverge from those in spinal tissue (Xuan et al. 2019; Zheng et al. 2021). At the oligodendrocyte level, OPCs from brain and spinal cord are transcriptionally distinct, with cholesterol biosynthesis/mammalian target of rapamycin (mTOR) signaling contributing more strongly to spinal precursor identity; hence, oligodendrocyte protection observed in cortical cultures may not fully predict spinal outcomes (Khandker et al. 2022). In addition to cell intrinsic differences, the extracellular constraints in SCI, like CSPG‐rich scarring and myelin‐associated inhibitors, are more severe and differently organized than in typical cortical injury paradigms, shaping axon growth and plasticity through pathways including Ras‐homologous A (RhoA)/ROCK and protein tyrosine phosphatase sigma (PTPσ) (Cafferty et al. 2010; Bradbury and Burnside 2019; Sami et al. 2020). Even among cortical regions, adult neurons exhibit heterogeneous morphology, growth, and firing properties, underscoring the need for cross‐system validation when extrapolating cortical findings to the spinal cord (van Niekerk et al. 2022). Accordingly, this cortical injury model should be regarded as a general mechanistic screening system to test whether Epac2 activation via S‐220 engages multiple CNS cell classes relevant to SCI. Notably, the biological plausibility of its relevance to SCI is supported by work showing that Epac2 activation with S‐220 overcomes CSPG inhibition, reduces glial activation, promotes axonal crossing in ex vivo spinal lesions, and improves locomotion when locally delivered after contusion SCI in adult rats (Guijarro‐Belmar, Viskontas, et al. 2019).

To address the direct relevance of this system for assessing SCI approaches, future work would need to directly assay nodes like phospho‐ERK1/2 (Thr202/Tyr204), phospho‐AKT (Ser473), and phospho‐CREB (Ser133) specifically relevant to neurons, oligodendrocyte‐lineage cells, astrocytes, microglia, and macrophages by quantitative Western blot with validated antibodies and transparent reporting, and evaluate transcriptional targets using reverse transcription‐quantitative polymerase chain reaction (RT‐qPCR).

Future work should also define the therapeutic window for Epac2 activation by delivering S‐220 across acute, subacute, and chronic phases, reflecting the time‐dependent pathobiology of SCI (Ahuja et al. 2017). Because timing influences outcomes clinically, they should vary start times and doses (Badhiwala et al. 2021). In the in vivo studies, follow‐up should extend to ≥ 12–16 weeks with quantitative readouts of survival, remyelination, gliosis, and axon growth to assess durability and safety (Ahuja et al. 2017). These would use standardized contusion/compression models with functional outcomes alongside histology.

## 5 Conclusions

In conclusion, the results in this present study provide compelling new support for the therapeutic promise of targeting Epac2 activation after CNS injury to promote repair. The combined neuroprotective and neuroregenerative effects seen with the Epac2 activation strategy in our study are on par with other promising CNS repair strategies reported in recent years, such as promoting SCI repair with parthenolide (Gaojian et al. 2020), or metformin ameliorating SCI‐induced neuronal apoptosis and promoting axon regeneration in the spinal cord (Wu et al. 2022), and, finally, promoting neuroprotection, neuroplasticity, and neurorecovery using a human anti‐RGMa monoclonal antibody Elezanumab (Jacobson et al. 2021), which have progressed into phase‐II clinical (NCT04295538). Future work should focus on validating these Epac2 activation effects with various molecular pathway assessments in vitro and testing Epac2 activation in vivo, assessing functional recovery in different clinically relevant animal models of SCI. Future studies may also include exploring the treatment time windows for Epac2 activation and the use of biomaterials to deliver S‐220. Overall, our study identifies Epac2 as a promising molecular target for the treatment of CNS injury.

## Author Contributions

**Hongming Ma:** conceptualization, data curation, formal analysis, funding acquisition, investigation, methodology, project administration, resources, software, validation, visualization, writing – original draft, writing – review and editing. **Guy S. Bewick:** project administration, supervision, visualization, writing – review and editing. **Wenlong Huang:** conceptualization, data curation, funding acquisition, methodology, project administration, resources, supervision, validation, visualization, writing – review and editing.

## Conflicts of Interest

The authors declare no conflicts of interest.

## Peer Review

The peer review history for this article is available at https://www.webofscience.com/api/gateway/wos/peer‐review/10.1111/ejn.70286.
